# Automated radiosynthesis of [^68^Ga]Ga‐PSMA‐11 and [^177^Lu]Lu‐PSMA‐617 on the iPHASE MultiSyn module for clinical applications

**DOI:** 10.1002/jlcr.3889

**Published:** 2020-11-02

**Authors:** Christian W. Wichmann, Uwe Ackermann, Stan Poniger, Kenneth Young, Benjamin Nguyen, Gordon Chan, John Sachinidis, Andrew M. Scott

**Affiliations:** ^1^ Tumor Targeting Laboratory Olivia Newton‐John Cancer Research Institute Heidelberg Victoria Australia; ^2^ School of Cancer Medicine La Trobe University Bundoora Victoria Australia; ^3^ Department of Molecular Imaging and Therapy Austin Health Heidelberg Victoria Australia; ^4^ Department of Medicine University of Melbourne Parkville Victoria Australia

**Keywords:** clinical quality control, Ga‐68, iPHASE MultiSyn, prostate cancer, PSMA‐11, PSMA‐617, theranostics

## Abstract

Prostate‐specific membrane antigen (PSMA)‐targeted imaging and therapy of prostate cancer using theranostic pairs is rapidly changing clinical practice. To facilitate clinical trials, fully automated procedures for the radiosyntheses of [^68^Ga]Ga‐PSMA‐11 and [^177^Lu]Lu‐PSMA‐617 were developed from commercially available precursors using the cassette based iPHASE MultiSyn module. Formulated and sterile radiopharmaceuticals were obtained in 76 ± 3% (*n* = 20) and 91 ± 4% (*n* = 15) radiochemical yields after 17 and 20 min, respectively. Radiochemical purity was always >95% and molar activities exceeded 792 ± 100 and 88 ± 6 GBq/μmol, respectively. Quality control showed conformity with all relevant release criteria and radiopharmaceuticals were used in the clinic.

## Graphical abstract

Methods for the fully automated production of a relevant theranostic pair of prostate‐specific membrane antigen (PSMA)‐targeting molecules are described. Quality control parameters and test results for clinical application are presented and the performance of the procedures is compared other reported protocols.

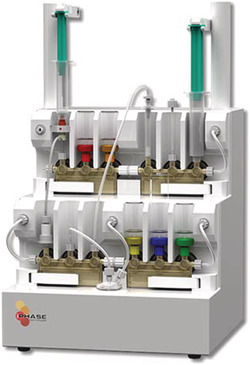

## 1 INTRODUCTION

Prostate‐specific membrane antigen (PSMA) is highly expressed in primary and metastatic lesions of prostate cancer.^1^ Due to its low abundance in healthy tissues, PSMA targeting with peptides allows for highly targeted delivery with demonstrated accuracy for diagnosis of primary and metastatic prostate cancer, and for therapeutic indications.^2^, ^3^, ^4^, ^5^, ^6^, ^7^ A number of small molecules targeting the extracellular domain of PSMA with exceptional affinity and specificity have been developed.^8^, ^9^, ^10^ More recently, this has led to the development of diagnostic and therapeutic pairs of PSMA‐targeting ligands. Although not a theranostic pair as per the definition, [^68^Ga]Ga‐PSMA‐11 and [^177^Lu]Lu‐PSMA‐617 are commonly used in this capacity and are under investigation in single site and multicentre clinical trials such as the TheraP and VISION studies.^11^, ^12^, ^13^, ^14^, ^15^, ^16^

Protocols for the manual, semiautomatic and fully automatic production of [^68^Ga]Ga‐PSMA‐11 have been published with overall radiochemical production yields for manual and semiautomatic processes generally >90%^17^, ^18^, ^19^, ^20^, ^21^, ^22^ and fully automated procedures including product formulation ranging between 57‐80% decay‐corrected (d.c.) radiochemical yield.^23^, ^24^, ^25^, ^26^, ^27^, ^28^ Manual and fully automated production of [^177^Lu]Lu‐PSMA‐617 has been reported in >90% radiochemical yields.^29^, ^30^, ^31^, ^32^

In order to facilitate PSMA clinical trials at Austin Health (Heidelberg, Australia), the radiosyntheses and formulation of [^68^Ga]Ga‐PSMA‐11 and [^177^Lu]Lu‐PSMA‐617 were automated and validated using an iPHASE (Melbourne, Australia) MultiSyn module.

## 2 EXPERIMENTAL

### 2.1 General methods

PSMA‐11 was purchased from ABX (Radeberg, Germany); sodium acetate (99%), ultratrace water (for ultratrace analysis) and hydrochloric acid (37% w/w, 99.999% trace metal basis) were purchased from Sigma‐Aldrich (Sydney, Australia); ethanol absolute (Emsure®) was purchased from Merck (Darmstadt, Germany); and saline (0.9% w/v) and water for injection (WFI) were purchased from Pfizer (NY, USA). Solutions of sodium acetate (0.25 M) and hydrochloric acid (0.1 M) were prepared from their respective stocks using ultratrace water and stored in containers at room temperature. PSMA‐11 precursor used for radiolabelling was prepared by dissolving PSMA‐11 (0.5 mg) in ultratrace water (2 ml). Aliquots of PSMA‐11 precursor solution (50 μl) were pipetted into 2‐ml Eppendorf tubes and stored at −4°C. Strata‐X solid‐phase extraction (SPE) cartridges were purchased from Phenomenex (Melbourne, Australia).

GMP grade PSMA‐617 was purchased from ABX (Radeberg, Germany). Lu‐PSMA‐617 high‐performance liquid chromatography (HPLC) standard was purchased from Endocyte (IN, USA). Sodium acetate, gentisic acid and sodium ascorbate were purchased from Sigma‐Aldrich (Sydney, Australia). A sterile stock solution of sodium acetate (0.4 M) was prepared, and aliquots were stored in sterile glass vials at room temperature. Standard hospital issue 0.22‐μm sterile filters, needles and WFI were used. Sterile glass vials were purchased from Huayi Isotopes (Changshu, China).

Gallium‐68 was eluted from an Eckert & Ziegler IGG100 Gallium generator. No carrier added (n.c.a.) [^177^Lu]LuCl_3_ in 0.04 M HCl was purchased from the Australian Nuclear Science and Technology Organisation (ANSTO, Lucas Heights, Australia). Sterile cassette kits were purchased from iPHASE Technologies (Melbourne, Australia).

Thin‐layer chromatography (TLC) of labelled PSMA‐11 samples was performed using aluminium‐backed silica gel 60 F_254_ strips (Merck, Darmstadt, Germany) and a 1:1 (v/v) mixture of 1 M ammonium acetate and methanol as a mobile phase. Instant TLC (iTLC) of labelled PSMA‐617 samples was performed using glass microfibre iTLC‐SG chromatography paper strips (Agilent, CA, USA) and 1.9% disodium hydrogen citrate as a mobile phase. Reversed‐phase HPLC (RP‐HPLC) was performed on a Shimadzu LC‐20 equipped with a SPD‐20A UV‐Vis detector and a LabLogic Flow‐RAM scintillation detector using a Phenomenex Kinetex C18 5 μm 150 × 4.6 mm column. Mobile phases were aqueous 0.08% TFA (A), acetonitrile (B), aq. 0.1% TFA (C) and 0.1% TFA in acetonitrile (D). Ga‐PSMA‐11 samples were analysed using a gradient of 5–80% D in C (v/v) over 10 min at a flow rate of 1.5 ml/min. Lu‐PSMA‐617 samples were analysed using a gradient of 15–35% B in A (v/v) over 15 min followed by 35–95% B in A (v/v) over 1 min and maintained for 3 min at a flow rate of 0.9 ml/min. Formulated material was analysed using a Charles River bacterial endotoxin testing unit.

### 2.2 Automated production of [^68^Ga]Ga‐PSMA‐11

**FIGURE 1:**
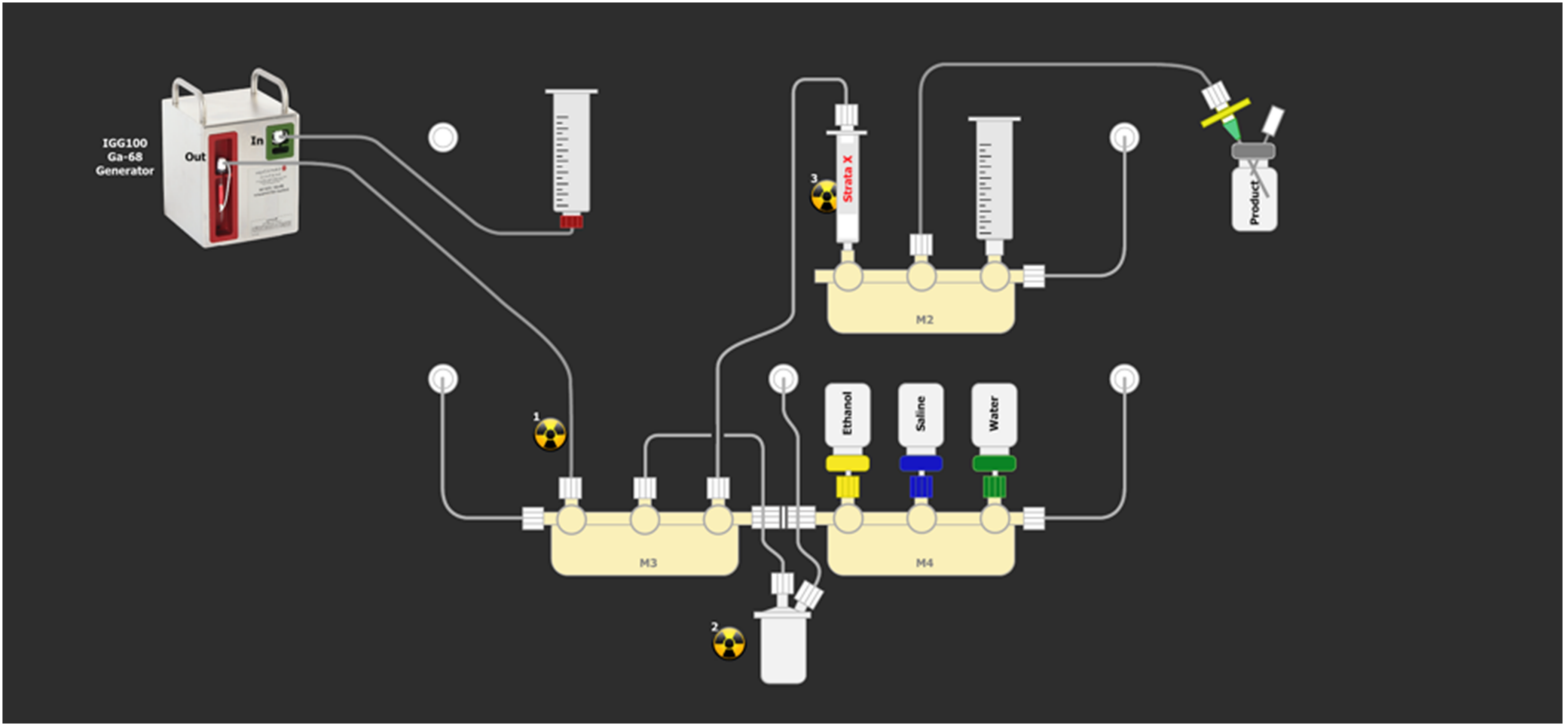
Schematic overview of iPHASE MultiSyn cassette and reagent set‐up for automated radiosynthesis of [^68^Ga]Ga‐PSMA‐11

Upon start‐up and installation of the hardware cassette excluding reagents, automated pressure testing was performed on all connections. The manifolds, reactor, SPE cartridge and reagent vial spikes were flushed with argon gas. Subsequently, reagents were installed as outlined in Figure 1. Vials containing ethanol (3.5 ml), saline (10 ml) and water (10 ml) were pushed onto their respective spikes on Manifold 4, and a solution of PSMA‐11 (10 μg, 9.88 nmol) in 0.25 M sodium acetate (1.6 ml) was added to the reactor via its centre port. A syringe containing 0.1 M HCl (5 ml) that was connected to the inlet of an Eckert & Ziegler IGG100 Gallium generator was placed in one of the syringe driver arms. The generator outlet was connected to Manifold 3. Finally, a 10‐ml glass vial equipped with a vented 0.22‐μm sterile filter was connected to manifold 2. The reagent vials were then pressurised with inert gas, and the SPE cartridge was conditioned with ethanol from the reagent vial (approximately 2 ml), washed with water (5 ml) and flushed dry. Ethanol (approximately 0.2 ml) was transferred into the reactor, and gallium‐68 was eluted from the generator using 0.1 M HCl in 0.3‐ml increments over 1 min of elution time. The first fraction (1.5 ml) was discarded, and the second fraction (3.5 ml) was collected into the preheated reactor. The reaction mixture was heated at 95°C for 5 min before being transferred over the SPE cartridge. Water (5 ml) was transferred first into the reactor and then loaded onto the SPE cartridge. The product was eluted from the SPE cartridge with ethanol (approximately 0.5 ml) using Syringe Driver 2 and diluted with saline (1.5 ml). The resulting solution was passed through a 0.22‐μm sterile filter and collected into the product vial. The reactor, SPE cartridge and transfer tubing from Manifold 2 to the product vial were flushed with further saline (5 ml) to complete formulation of the product.

### 2.3 Automated production of [^177^Lu]Lu‐PSMA‐617

Automated pressure testing of all connections was performed with the manifolds, Transfer Syringe 3 (Manifold Position 6) and the reactor installed, and the reactor was flushed with inert gas. Subsequently, reagent vials and syringes were installed as described in Table 1.

**TABLE 1:** Reagent set‐up for automated production of n.c.a. [^177^Lu]Lu‐PSMA‐617 using an iPHASE MultiSyn module

Location	Reagent/set‐up
G1	Argon gas supply to manifold M1
W2	Liquid waste out from manifold M2 and argon/vacuum supply
R	Reactor vent with argon/vacuum supply
Manifold Position 1^SD^	Syringe 1 (1 ml) containing PSMA‐617 (125 μg, 103 nmol) + 4‐mg gentisic acid in 1‐ml 0.4 M sodium acetate
Manifold Position 2	Line to Lu‐177 vial with PEEK needle and 19 G vent needle
Manifold Position 3	Syringe 2 (10 ml) containing 500‐mg sodium ascorbate + 1‐mg DTPA in 10‐ml water for injection
Manifold Position 4	Line to reactor vial
Manifold Position 5	Line to product vial (20 ml) with 0.22‐μm vented sterile filter + 21 G needle and 19 G vent needle
Manifold Position 6^SD^	Syringe 3 (10 ml, empty)

*Note:* Manifold Positions 1 and 6 are equipped with syringe drivers (SD).

[^177^Lu]LuCl_3_ in 0.04 M HCl (approximately 1 ml) was transferred from the Lu‐177 vial into the reactor. One millilitre of a 0.4 M sodium acetate solution containing PSMA‐617 (125 μg, 103 nmol) and 4‐mg gentisic acid was transferred from Syringe 1 into the Lu‐177 vial, remained there for 10 s and was transferred into the preheated reactor vial. After flushing the transfer line with inert gas, the reaction was heated to 95°C for 15 min. During the labelling reaction, half of the contents in Syringe 2, an aqueous solution containing 500‐mg sodium ascorbate and 1‐mg DTPA in 10‐ml WFI, were drawn into Syringe 3. At the conclusion of the reaction, the reactor vial was cooled to 40°C and the contents in Syringe 3 were transferred into the reactor vial. The reaction mixture was drawn into Syringe 3 and passed through a vented 0.22‐μm sterile filter into the product vial. The reactor vial was then flushed with the remaining sodium ascorbate/DTPA solution in Syringe 2 (approximately 5 ml), and the solution was transferred into the product vial via Syringe 3. Finally, the transfer line and sterile filter leading to the product vial were flushed with inert gas.

## 3 RESULTS AND DISCUSSION

### 3.1 Development of automated [^68^Ga]Ga‐PSMA‐11 synthesis protocol

Automated radiosynthesis of [^68^Ga]Ga‐PSMA‐11 was achieved on an iPHASE MultiSyn module using the set‐up outlined in Figure 1. Gallium‐68 was obtained from an Eckert & Ziegler IGG100 Gallium generator by elution with 5‐ml 0.1 M hydrochloric acid. The first fraction (1.5 ml) was automatically discarded to reduce germanium‐68 content and elution volume to enable easier buffering. The second fraction (3.5 ml) containing Ga‐68 was collected into the reactor vial and used without prepurification or preconcentration. The reactor was charged with a small amount of ethanol prior to addition of the radioisotope to moderate radiolytic breakdown of reagents. Addition of the second generator fraction to the reactor vial containing PSMA‐11 in sodium acetate resulted in a reaction pH of 4. Purification and reformulation of [^68^Ga]Ga‐PSMA‐11 was performed using a polymer‐based SPE cartridge (Phenomenex Strata‐X) before being sterile filtered into a sterile product collection vial (Figure S1 a‐d).

**FIGURE 2:**
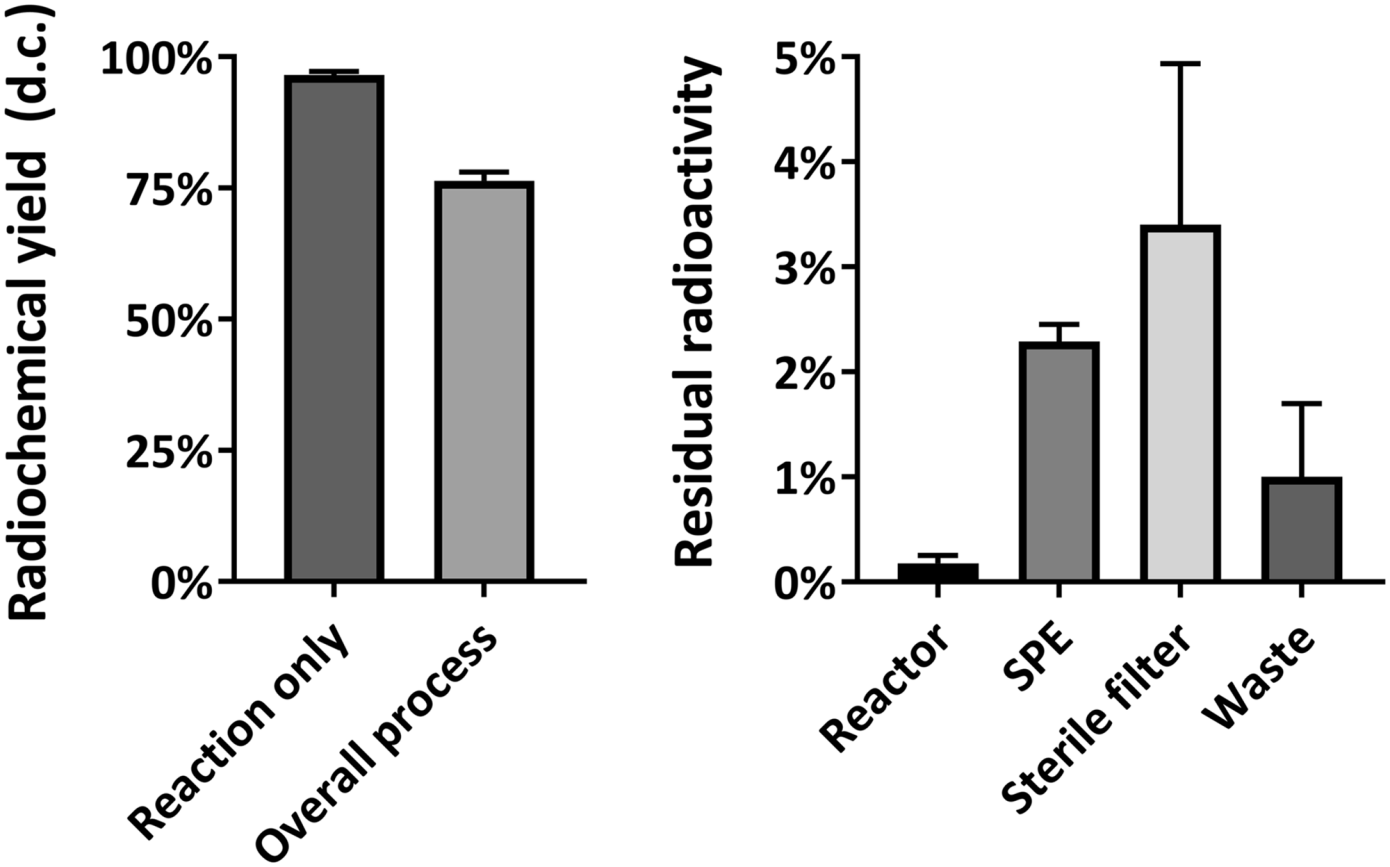
Radiochemical reaction and overall process yield of the automated synthesis of [^68^Ga]Ga‐PSMA‐11 (left). Residual radioactivity (*n* = 8) in the reactor vial, Strata‐X solid‐phase extraction (SPE) cartridge, sterile filter and waste (right). Data shown as mean ± SD

Complexation of Ga‐68 by PSMA‐11 proceeded reproducibly in 96.6 ± 0.6% (*n* = 8) radiochemical yield (Figure 2, left). Overall, the process yielded [^68^Ga]Ga‐PSMA‐11 in 76.2 ± 3.4% (*n* = 20) radiochemical yield after 17 min. Residual activity in the reactor vial was negligible at 0.18 ± 0.08% (Figure 2, right). Residual activity on SPE cartridge and sterile filter accounted for the majority of losses with 2.3 ± 0.2% and 3.4 ± 1.5%, respectively. The waste contained 1.0 ± 0.7% of the total activity eluted from the Ga‐68 generator.

### 3.2 Development of automated [^177^Lu]Lu‐PSMA‐617 synthesis protocol

**FIGURE 3:**
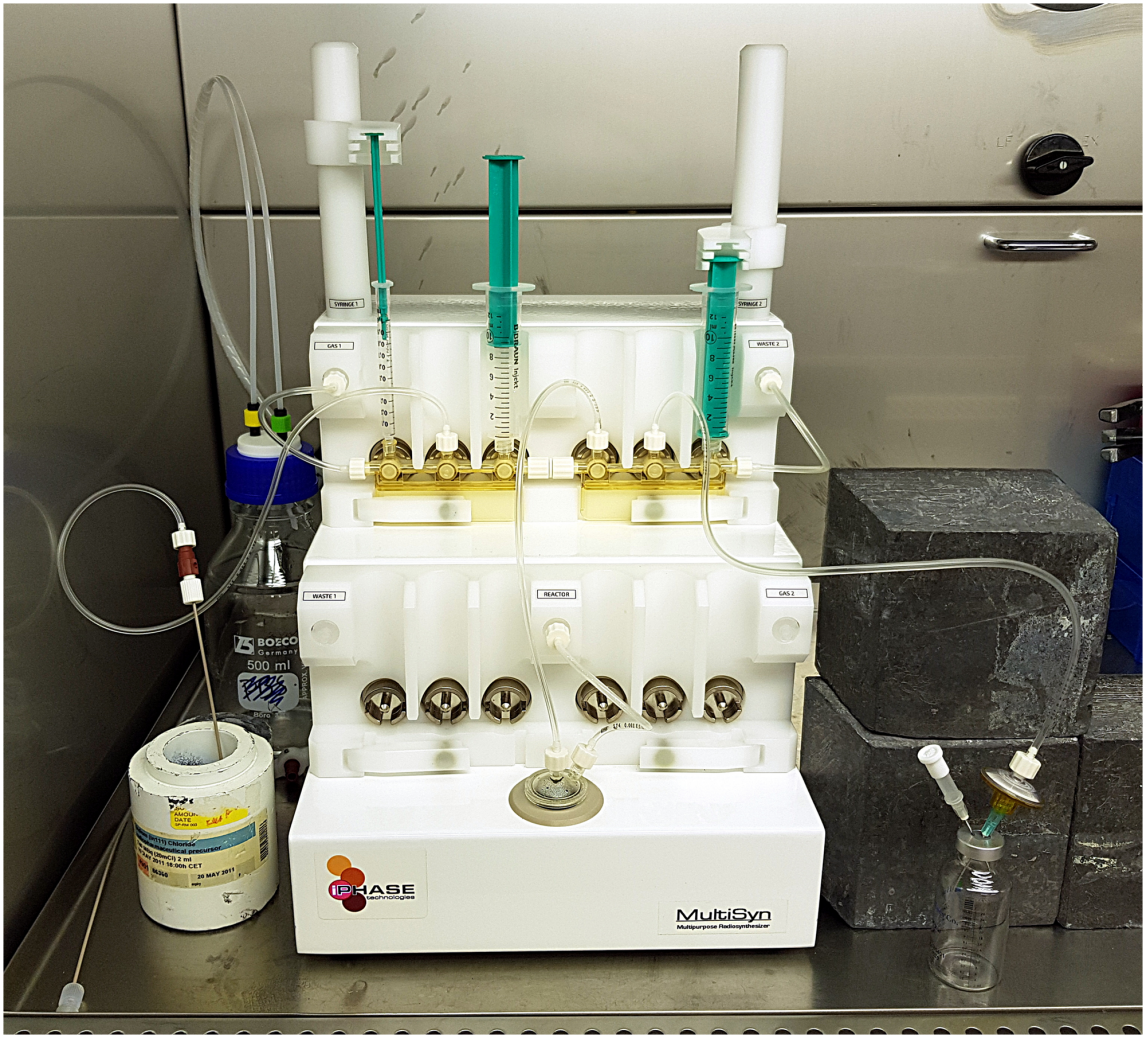
Picture of iPHASE MultiSyn set‐up for automated radiosynthesis of [^177^Lu]Lu‐PSMA‐617 according to Figure 4 and Table 1

**FIGURE 4:**
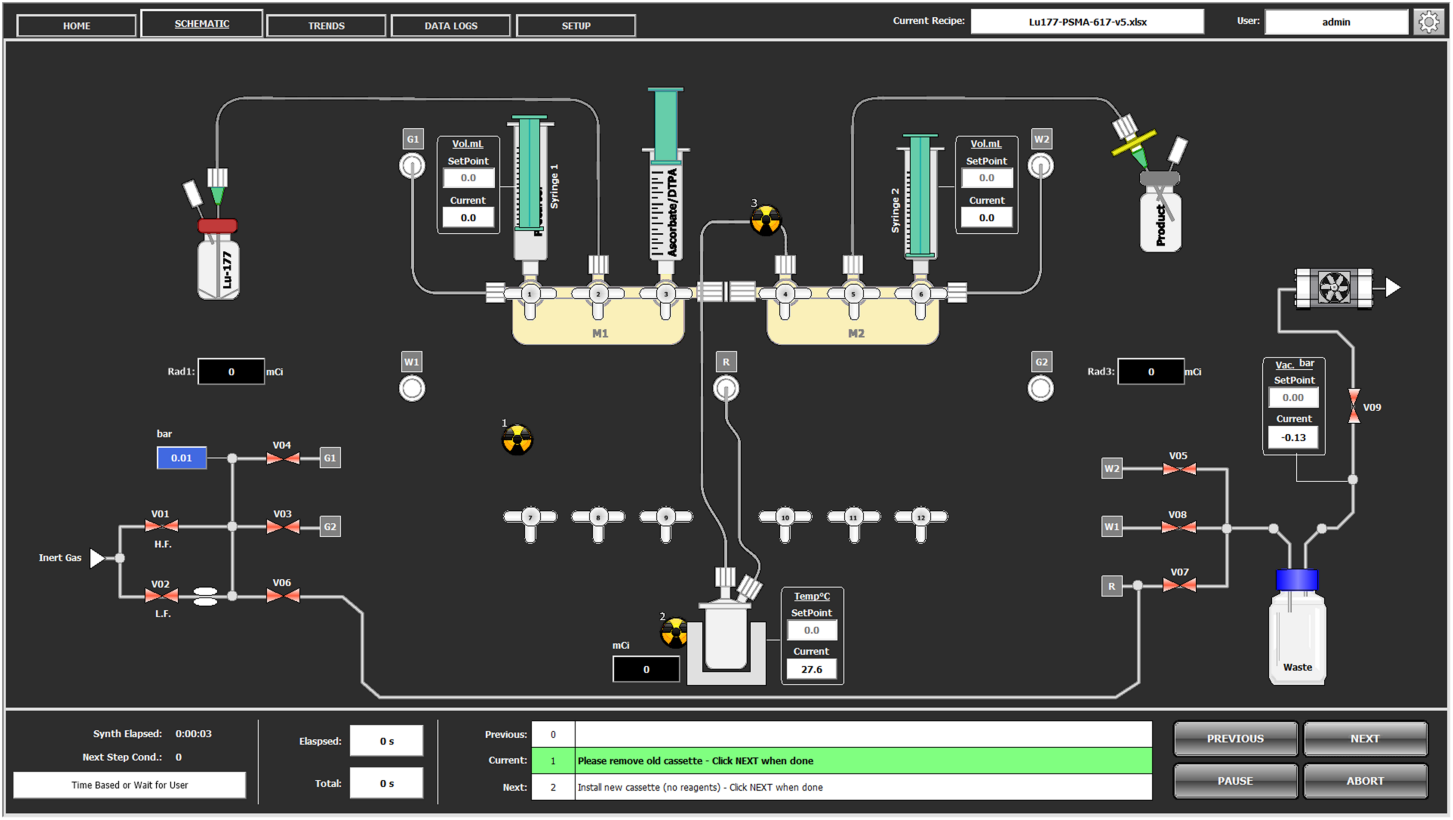
Schematic overview of iPhase MultiSyn cassette and reagent set‐up for radiosynthesis of [^177^Lu]Lu‐PSMA‐617 as described in Table 1

Quantitative complexation of ^177^Lu^3+^ with DOTA was achieved in 0.4 M sodium acetate solution at 95°C after 15 min at pH 4.5–5. The reaction was stabilised using gentisic acid, and the final product was formulated in sodium ascorbate containing DTPA. The process was automated using the cassette based iPHASE MultiSyn module (Figure 3). Fluid transfers were performed using either gas pressure/vacuum or the two available syringe drivers. Figure 4 shows a schematic overview of the installed synthesis cassette consisting of two manifolds, one reactor vial, three syringes and a product collection vial. The reagents and transfer lines were set up as described in Table 1.

**FIGURE 5:**
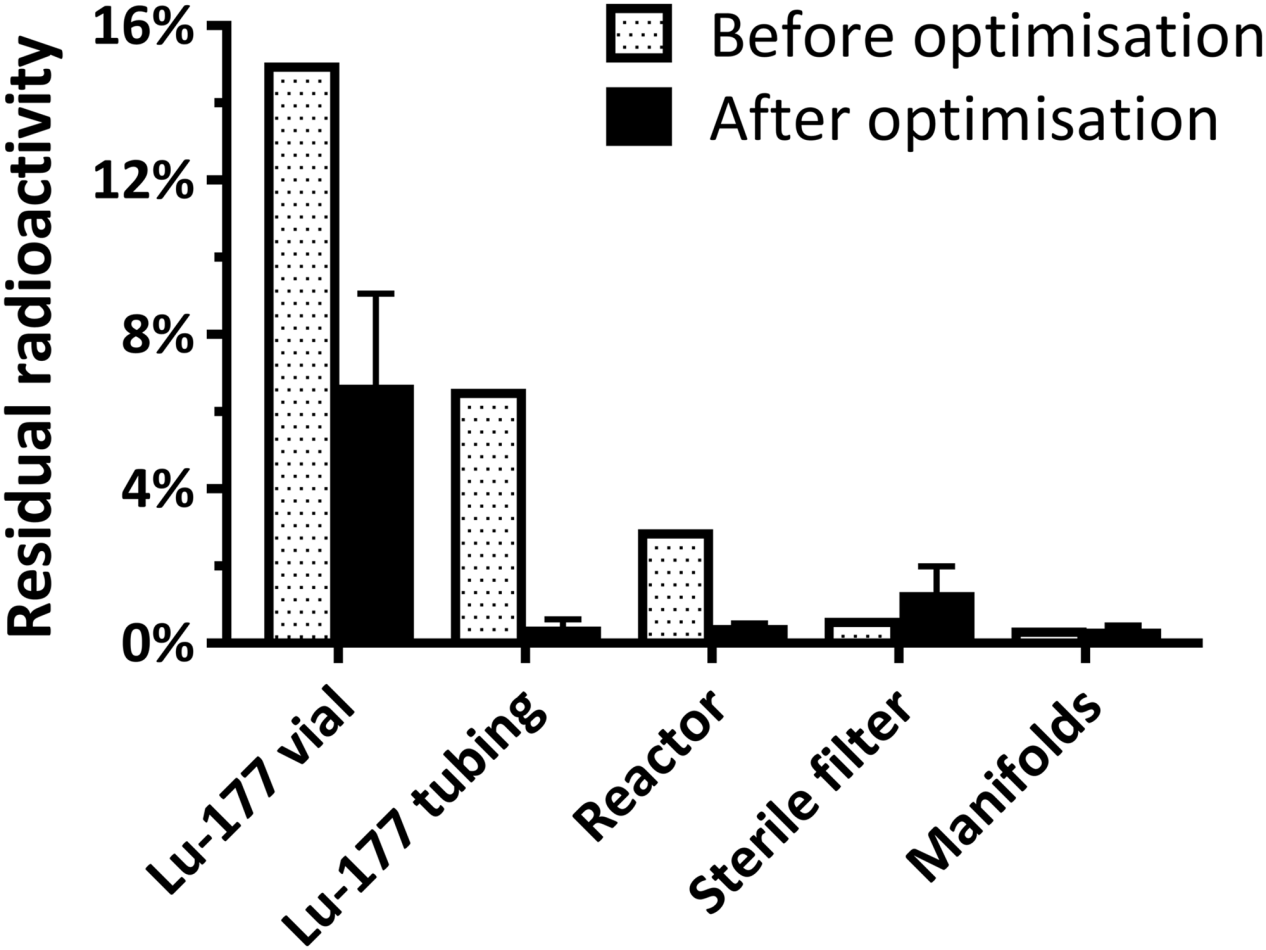
Residual Lu‐177 radioactivity before and after optimisation. Data shown as mean ± SD (*n* = 1–3)

Initially, we experienced large radioactivity losses due to residual [^177^Lu]LuCl_3_ remaining in the Lu‐177 vial (Figure 5). This was improved by flushing the Lu‐177 vial with the precursor solution following isotope transfer into the reactor. This led to improved Lu‐177 recovery in the reactor. Residual radioactivity remaining in the Lu‐177 vial decreased by 55% from 15.0% to 6.7 ± 1.8% (*n* = 3). Losses in the Lu‐177 vial transfer tubing were reduced by 94% from 6.6% to 0.4 ± 0.1% (*n* = 3).

Losses during the recovery of [^177^Lu]Lu‐PSMA‐617 from the reactor were addressed by performing a saline wash of the reactor during product formulation resulting in 0.5 ± 0.0% (*n* = 3) of radioactivity lost in the reactor. Residual radioactivity on the sterile filter and in the manifolds remained constant at 1.2 ± 0.5% (*n* = 4) and 0.4 ± 0.1% (*n* = 4), respectively. This resulted in an overall increase in radiochemical yield of [^177^Lu]Lu‐PSMA‐617 from 75% to 91.1 ± 3.8% (*n* = 14) at end of synthesis (EOS). The total synthesis time was 20 min including formulation and sterile filtration (Figure S3 a‐d).

### 3.3 Quality control for clinical use

Quality control was performed on [^68^Ga]Ga‐PSMA‐11 and [^177^Lu]Lu‐PSMA‐617 according to the criteria outlined in Table 2. The reported results were obtained from 12 and 15 clinical productions, respectively. Sterile filters were tested for membrane filter integrity which was always >50 psi. Where applicable, solvent analysis was performed which showed <10% ethanol.

**TABLE 2:** Quality control results of [^68^Ga]Ga‐PSMA‐11 (*n* = 12) and [^177^Lu]Lu‐PSMA‐617 (*n* = 15); molar activities at end of synthesis (EOS) reported

Test	[^68^Ga]Ga‐PSMA‐11	[^177^Lu]Lu‐PSMA‐617
Overall production yield (d.c.) (%)	76 ± 3	91 ± 4
Product volume (ml)	6.8 ± 0.2	9.4 ± 0.7
Molar activity (EOS) (GBq/μmol)	>792 ± 100	>88 ± 6
Radiochemical identity	RT within 10% of standard	RT within 10% of standard
Radiochemical purity (HPLC) (%)	99.9 ± 0.2	96.6 ± 0.7
Residual Ga‐68/Lu‐177 (HPLC) (%)	0.11 ± 0.18	0.89 ± 0.52
Residual Ga‐68 (ionic + colloidal)/Lu‐177 (TLC) (%)	0.60 ± 0.51	0.62 ± 0.23
Chemical purity (*m*_PSMA_) (μg)	<1.0	‐
Radionuclidic identity (T_1/2_) (min/day)	67.7 ± 2.5	6.45 ± 0.06
Radionuclidic identity (MCA) (keV)	512.3 ± 1.6	‐
Radionuclidic purity (%)	<0.001 Ge‐68	>99.9
Appearance	Clear, colourless, particulate free	Clear, colourless—Slightly yellow, particulate free
pH	7.0 ± 0.1	7.0 ± 0.0
Sterility test	No growth after 14 days	No growth after 14 days
Bacterial endotoxin test (EU/ml)	<2.5^a^	<2.5^a^

*Note:* Data shown as mean ± SD.

^a^ Limit of detection for bacterial endotoxins is 2.5 EU/ml.

Overall, production of [^68^Ga]Ga‐PSMA‐11 proceeded in 76% (d.c.) radiochemical yield providing the product in a volume of 6.8 ml of <10% ethanol in saline. HPLC analysis of residual PSMA‐11 showed <1.0 μg PSMA‐11 per production batch which results in molar activities of at least 792 ± 100 GBq/μmol (EOS). Radiochemical purity via HPLC was always >99% (both diastereomers combined) and free ionic (HPLC) and combined ionic/colloidal Ga‐68 (TLC, *R*_f_ = 0.0–0.1) were always <1.0% and <3.2%, respectively (Figure S2 a‐d).

Production of [^177^Lu]Lu‐PSMA‐617 proceeded in 91% (d.c.) radiochemical yield providing the product in a volume of 9.4 ml. Molar activity was not a mandatory release criterion for [^177^Lu]Lu‐PSMA‐617. Based on the starting amount of PSMA‐617 (103 nmol) and the radiochemical purity, the molar activity was estimated to be ≥88 ± 6 GBq/μmol (EOS). Radiochemical purity by HPLC was always ≥95% and unreacted lutetium‐177 (iTLC, *R*_f_ = 0.9–1.0) was always ≤2.4% (Figure S4 a‐d).

Radionuclidic identity and purity were confirmed for both constructs. Sterility and bacterial endotoxin testing showed no positive results over the limit of detection. All productions fulfilled the quality control release criteria and were used in the clinic.

## 4 CONCLUSIONS

In summary, the syntheses of a diagnostic and therapeutic pair of PSMA‐targeting agents were automated using the same commercially available synthesiser. Production yields were comparable with other reported fully automated procedures. Both products passed quality control criteria without fail and are being used in clinical trials at the Austin Hospital.

## Supporting information




**Figure S1** a: Radioactivity detector traces of [^68^Ga]Ga‐PSMA‐11 radiosynthesis on iPHASE MultiSyn.
Figure S1 b: Temperature traces of [^68^Ga]Ga‐PSMA‐11 radiosynthesis on iPHASE MultiSyn.
Figure S1 c: Argon gas pressure trace of [^68^Ga]Ga‐PSMA‐11 radiosynthesis on iPHASE MultiSyn.
Figure S1 d: Vacuum traces of [^68^Ga]Ga‐PSMA‐11 radiosynthesis on iPHASE MultiSyn.
Figure S2 a: Radio‐HPLC trace of [^68^Ga]Ga‐PSMA‐11 (both diastereomers).
Figure S2 b: HPLC trace of Ga‐PSMA‐11 standard.
Figure S2 c: TLC trace of [^68^Ga]Ga‐PSMA‐11 (R_f_ = 0.8‐1.0). Free and colloidal Ga‐68 (R_f_ = 0‐0.2)
Figure S3 a: Radioactivity detector traces of [^177^Lu]Lu‐PSMA‐617 radiosynthesis on iPHASE MultiSyn.
Figure S3 b: Temperature traces of [^177^Lu]Lu‐PSMA‐617 radiosynthesis on iPHASE MultiSyn.
Figure S3 c: Argon gas pressure trace of [^177^Lu]Lu‐PSMA‐617 radiosynthesis on iPHASE MultiSyn.
Figure S3 d: Vacuum traces of [^177^Lu]Lu‐PSMA‐617 radiosynthesis on iPHASE MultiSyn.
Figure S4 a: Radio‐HPLC trace of [^177^Lu]Lu‐PSMA‐617.
Figure S4 b: HPLC trace of [^177^Lu]Lu‐PSMA‐617.
Figure S4 c: HPLC trace of Lu‐PSMA‐617 standard.
Figure S4 d: iTLC trace of [^177^Lu]Lu‐PSMA‐617 (Rf = 0.3). Free and DTPA‐bound Lu‐177 (R_f_ = 0.9‐1.0).
