# Supplementary material for: Automated radiosynthesis of [68Ga]Ga‐PSMA‐11 and [177Lu]Lu‐PSMA‐617 on the iPHASE MultiSyn module for clinical applications
Source: J Labelled Comp Radiopharm. 2020 Nov 2;64(3):140–6. doi: 10.1002/jlcr.3889 (PMC8048907; doi:10.1002/jlcr.3889)
Supplement: Supplementary file 1 — Figure S1 a: Radioactivity detector traces of [68Ga]Ga‐PSMA‐11 radiosynthesis on iPHASE MultiSyn. Figure S1 b: Temperature traces of [68Ga]Ga‐PSMA‐11 radiosynthesis on iPHASE MultiSyn. Figure S1 c: Argon gas pressure trace of [68Ga]Ga‐PSMA‐11 radiosynthesis on iPHASE MultiSyn. Figure S1 d: Vacuum traces of [68Ga]Ga‐PSMA‐11 radiosynthesis on iPHASE MultiSyn. Figure S2 a: Radio‐HPLC trace of [68Ga]Ga‐PSMA‐11 (both diastereomers). Figure S2 b: HPLC trace of Ga‐PSMA‐11 standard. Figure S2 c: TLC trace of [68Ga]Ga‐PSMA‐11 (Rf = 0.8‐1.0). Free and colloidal Ga‐68 (Rf = 0‐0.2) Figure S3 a: Radioactivity detector traces of [177Lu]Lu‐PSMA‐617 radiosynthesis on iPHASE MultiSyn. Figure S3 b: Temperature traces of [177Lu]Lu‐PSMA‐617 radiosynthesis on iPHASE MultiSyn. Figure S3 c: Argon gas pressure trace of [177Lu]Lu‐PSMA‐617 radiosynthesis on iPHASE MultiSyn. Figure S3 d: Vacuum traces of [177Lu]Lu‐PSMA‐617 radiosynthesis on iPHASE MultiSyn. Figure S4 a: Radio‐HPLC trace of [177Lu]Lu‐PSMA‐617. Figure S4 b: HPLC trace of [177Lu]Lu‐PSMA‐617. Figure S4 c: HPLC trace of Lu‐PSMA‐617 standard. Figure S4 d: iTLC trace of [177Lu]Lu‐PSMA‐617 (Rf = 0.3). Free and DTPA‐bound Lu‐177 (Rf = 0.9‐1.0). [file JLCR-64-140-s001.docx]

**Supplementary Material**

**Automated radiosynthesis of [^68^Ga]Ga-PSMA-11 and [^177^Lu]Lu-PSMA-617 on the iPHASE MultiSyn module for clinical applications**

Christian W. Wichmann^1,2^, Uwe Ackermann^1,3,4^, Stan Poniger^3^, Kenneth Young^3^, Benjamin Nguyen^3^, Gordon Chan^3^, John Sachinidis^3^, and Andrew M. Scott^1-4^.

^*^ Correspondence: [christian.wichmann@onjcri.org.au](mailto:christian.wichmann@onjcri.org.au)

1. Olivia Newton-John Cancer Research Institute, Heidelberg, VIC 3084, Australia

2. School of Cancer Medicine, La Trobe University, Bundoora, VIC 3083, Australia

3. Department of Molecular Imaging and Therapy, Austin Health, Level 1 HSB, 145 Studley Road, Heidelberg, VIC 3084, Australia

4. Department of Medicine, University of Melbourne, Parkville, VIC 3050, Australia

**Figure S1 a:** Radioactivity detector traces of [^68^Ga]Ga-PSMA-11 radiosynthesis on iPHASE MultiSyn.


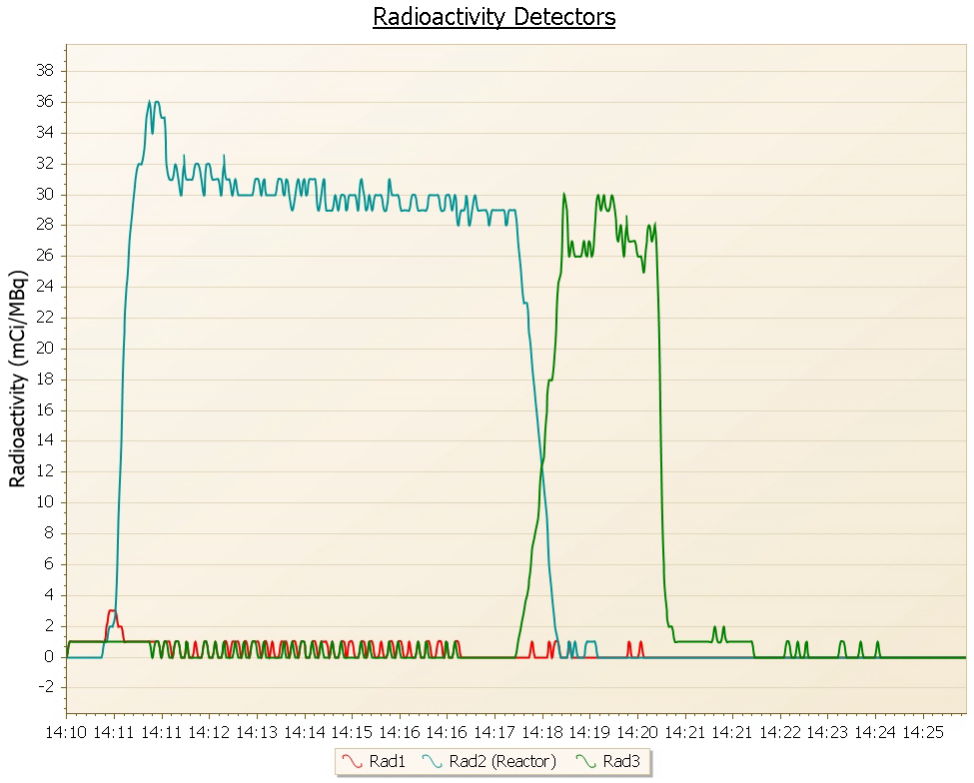


**Figure S1 b:** Temperature traces of [^68^Ga]Ga-PSMA-11 radiosynthesis on iPHASE MultiSyn.


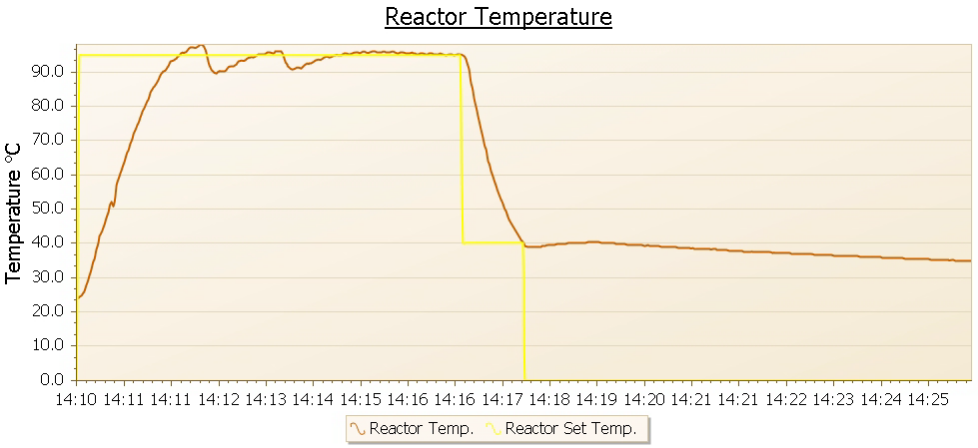


**Figure S1 c:** Argon gas pressure trace of [^68^Ga]Ga-PSMA-11 radiosynthesis on iPHASE MultiSyn.


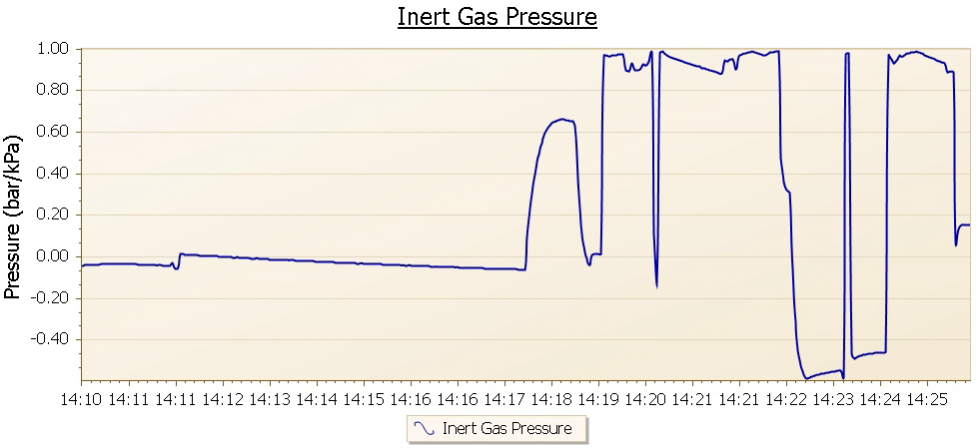


**Figure S1 d:** Vacuum traces of [^68^Ga]Ga-PSMA-11 radiosynthesis on iPHASE MultiSyn.


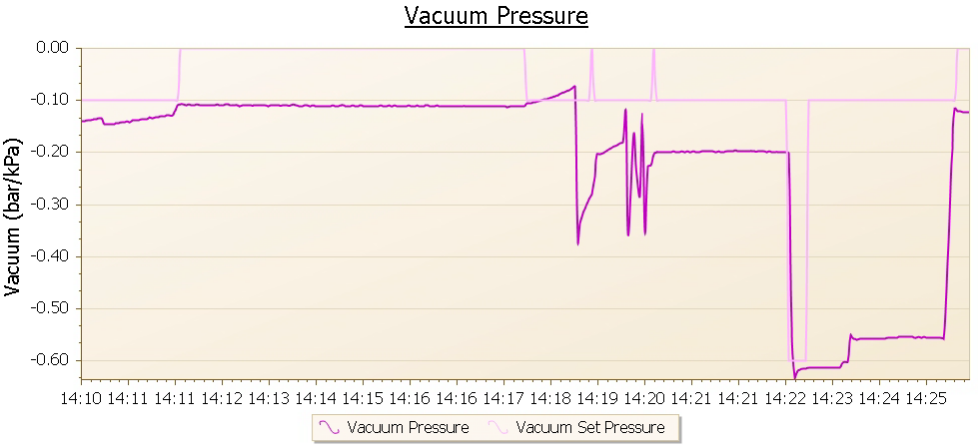


**Figure S2 a:** Radio-HPLC trace of [^68^Ga]Ga-PSMA-11 (both diastereomers).


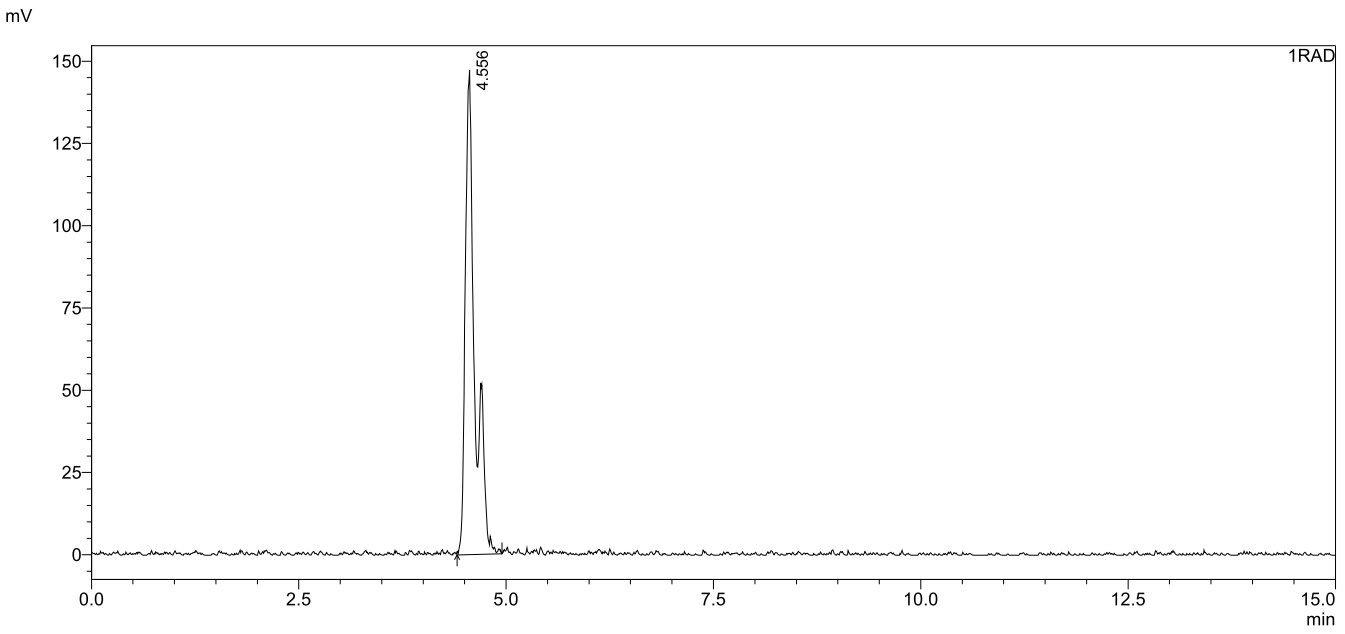


**Figure S2 b:** HPLC trace of Ga-PSMA-11 standard.


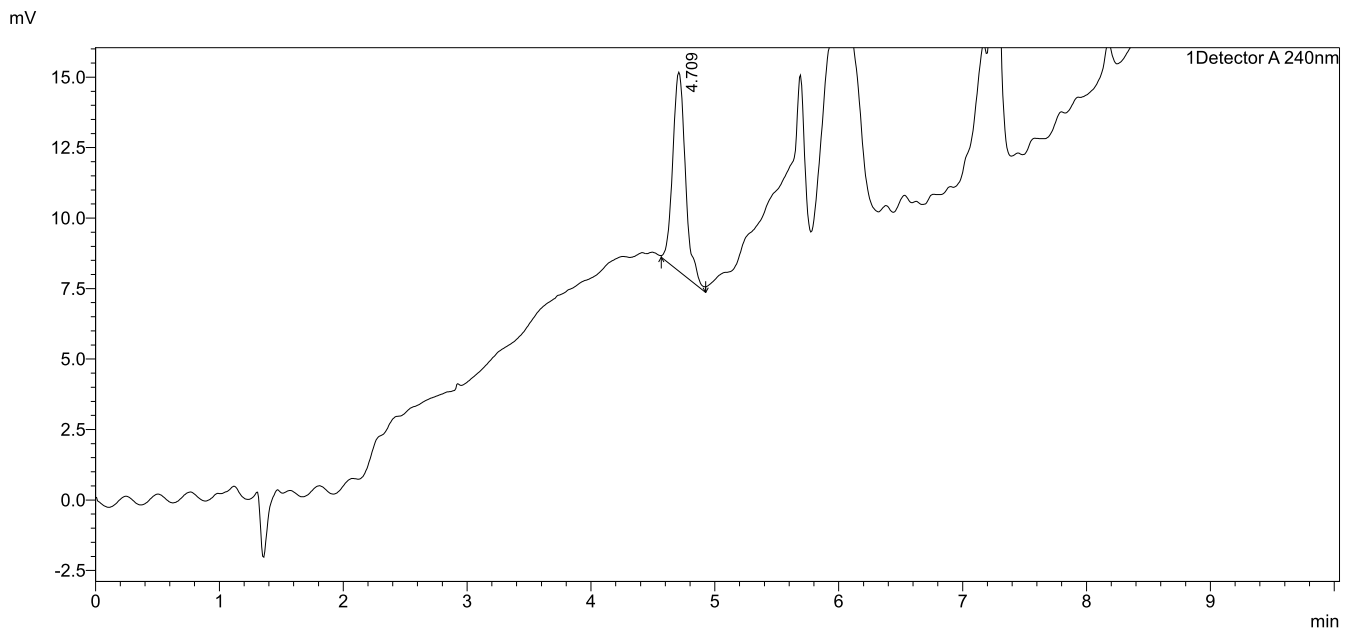


**Figure S2 c:** TLC trace of [^68^Ga]Ga-PSMA-11 (R_f_ = 0.8 - 1.0). Free and colloidal Ga-68 (R_f_ = 0 - 0.2)

**Figure S3 a:** Radioactivity detector traces of [^177^Lu]Lu-PSMA-617 radiosynthesis on iPHASE MultiSyn.


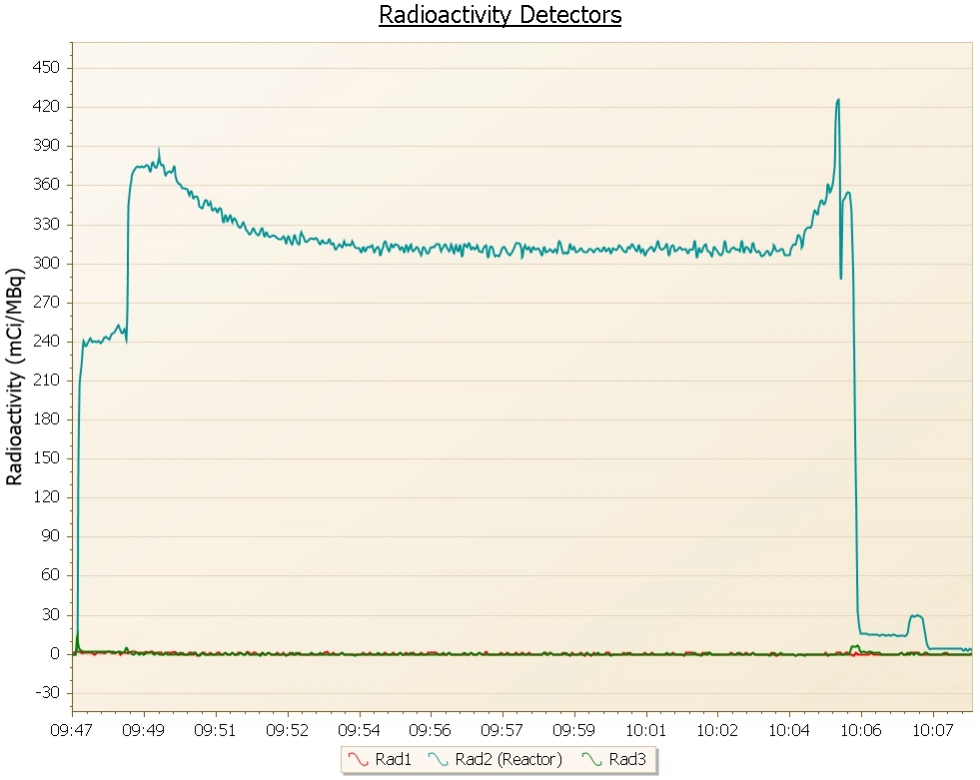


**Figure S3 b:** Temperature traces of [^177^Lu]Lu-PSMA-617 radiosynthesis on iPHASE MultiSyn.


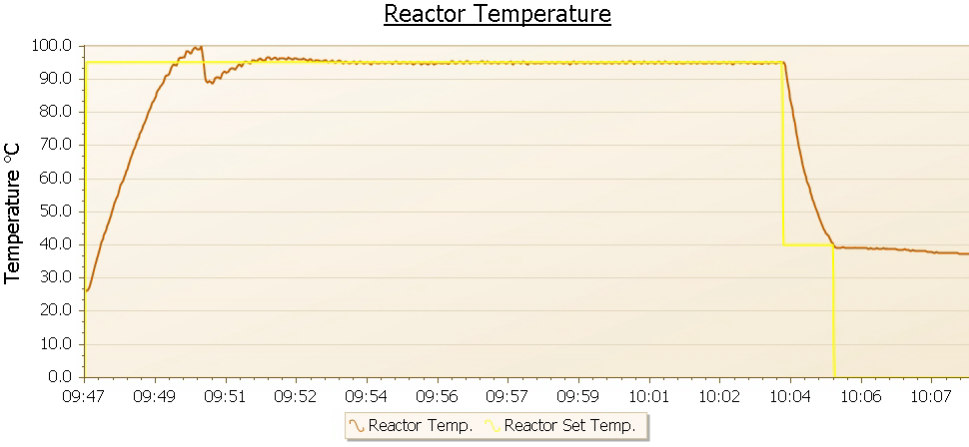


**Figure S3 c:** Argon gas pressure trace of [^177^Lu]Lu-PSMA-617 radiosynthesis on iPHASE MultiSyn.


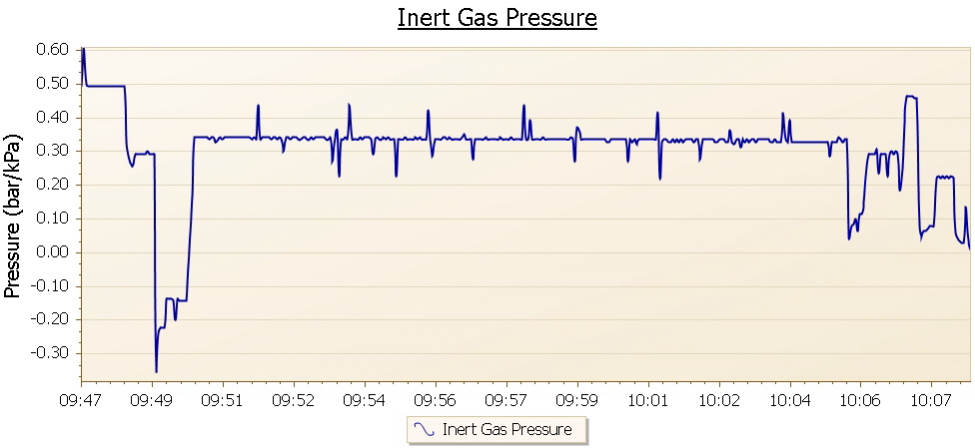


**Figure S3 d:** Vacuum traces of [^177^Lu]Lu-PSMA-617 radiosynthesis on iPHASE MultiSyn.


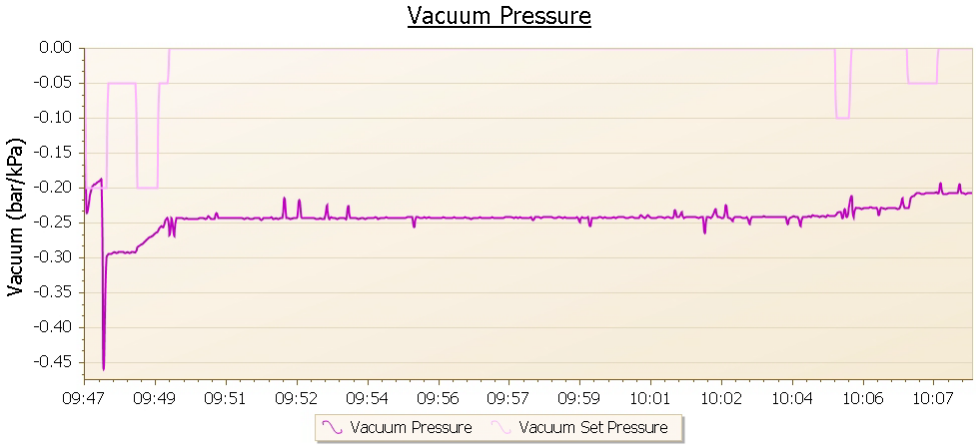


**Figure S4 a:** Radio-HPLC trace of [^177^Lu]Lu-PSMA-617.


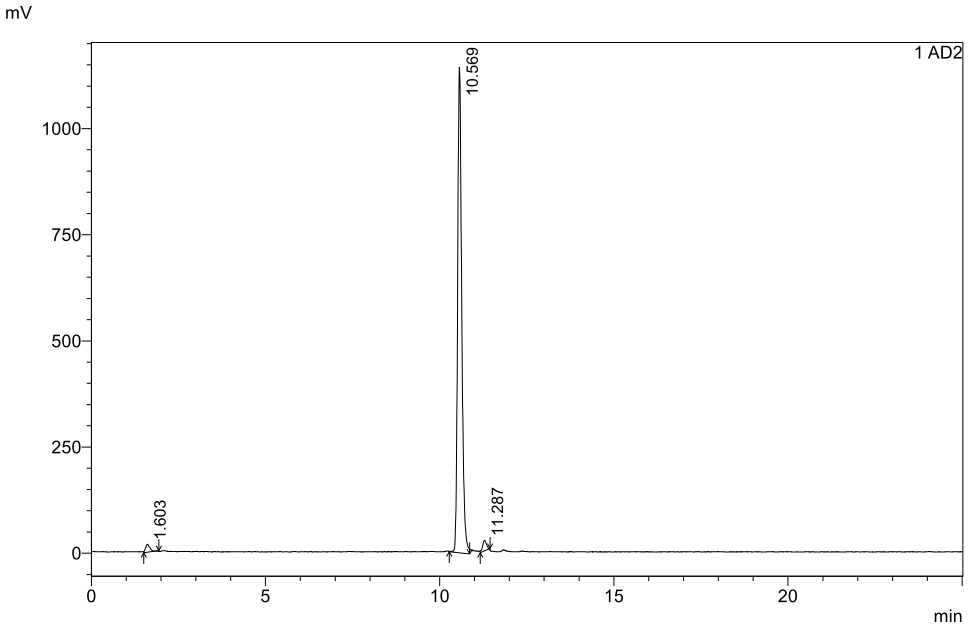


**Figure S4 b:** HPLC trace of [^177^Lu]Lu-PSMA-617.


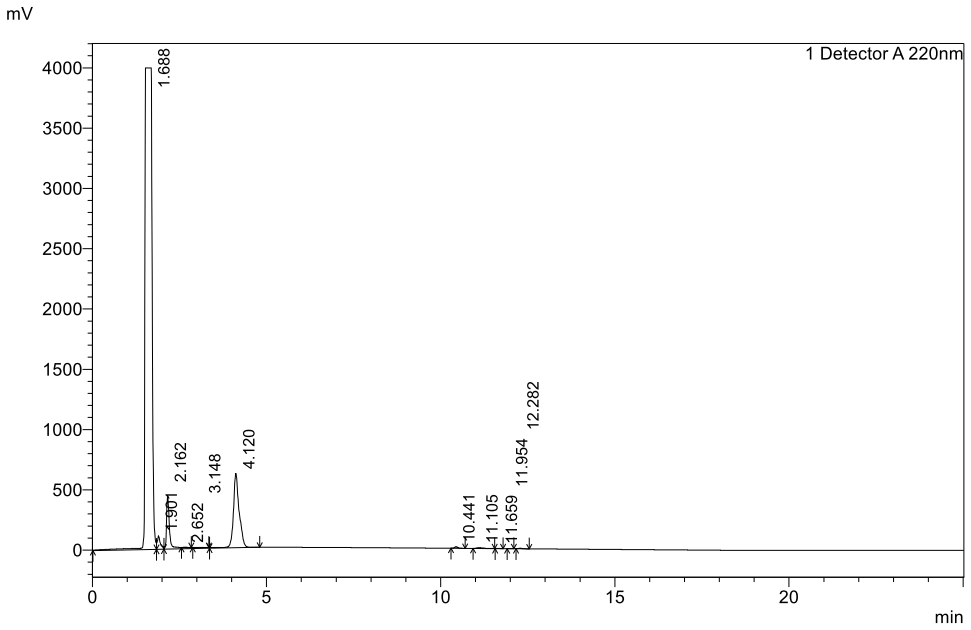


**Figure S4 c:** HPLC trace of Lu-PSMA-617 standard.

**Figure S4 d:** iTLC trace of [^177^Lu]Lu-PSMA-617 (Rf = 0.3). Free and DTPA-bound Lu-177 (R_f_ = 0.9 - 1.0).
